# Dietary urea levels induce morphological damage of cells, lipid metabolism disorders, and ammonia accumulation in subcutaneous fat in Tibetan sheep

**DOI:** 10.3389/fvets.2026.1887140

**Published:** 2026-07-31

**Authors:** Rongfu Song, Juyuan He, Anum Ali Ahmad, Linsheng Gui, Shengzhen Hou, Chao Yang

**Affiliations:** 1 Key Laboratory of Plateau Ecology and Agriculture, Qinghai University, Xining, China; 2 College of Agriculture and Animal Husbandry, Qinghai University, Xining, China; 3 The Roslin Institute, The University of Edinburgh, Easter Bush Campus, Edinburgh, Midlothian, United Kingdom

**Keywords:** lipidomics, subcutaneous fat, Tibetan sheep, urea, volatile flavor compounds

## Abstract

**Introduction:**

Urea shows substantial potential as a cost-effective alternative to soybean meal in ruminant production. Given the critical role of subcutaneous adipose tissue (SAT) in meat quality, this study evaluated the effects of different dietary urea levels on the histomorphology, fatty acid composition, lipidomic profiles, and volatile flavour compounds of SAT in Tibetan sheep.

**Methods:**

Eighty female lambs (16.2 ± 1.01 kg, 2 months old) were randomly assigned to four dietary treatments supplemented with 0% (U0), 0.80% (U1), 1.19% (U2), and 1.61% (U3) urea. The 105-day trial comprised a 15-day adaptation period followed by a 90-day experimental period.

**Results:**

The results indicated that: high urea levels (U2 and U3) disrupted adipocyte integrity, resulting in disordered arrangement, cellular rupture, and a significant reduction in adipocyte diameter (*p* < 0.05). Increasing dietary urea levels significantly decreased the concentrations of trans monounsaturated fatty acids (e.g., C16:1 T) and n-3 polyunsaturated fatty acids (*p* < 0.05), while increasing the level of cis monounsaturated fatty acid C18:1n9c (*p* < 0.05). Lipidomic analysis identified 48 differential lipid molecules (|log2Fold change| ≥ 2, adjusted *p* < 0.05), primar ily comprising glycerolipids (Triglycerides, Triglycerides) and glycerophospho lipids (phosphatidylcholine, phosphatidylethanolamine, Phosphatidylglycerol). Urea supplementation significantly influenced the volatile compounds profile (*p* < 0.05); excessive urea (U3) led to ammonia accumulation and pungent off odors, whereas moderate supplementation imparted desirable milky and vanilla aromas.

**Discussion:**

In conclusion, dietary urea levels affected lipid metabolism and reduced trans-fatty acid content; excessive dietary urea disrupted cell structure, increased ammonia accumulation and reduced aroma-contributing substances in the SAT of Tibetan sheep.

## 1 Introduction

Tibetan sheep are a characteristic and valuable livestock breed of the Qinghai-Tibetan Plateau, well adapted to extreme environments such as high altitude, hypoxia, cold stress, and seasonal nutritional scarcity (1). They serve as a primary source of livelihood for local herdsmen. The Qinghai-Tibet Plateau is characterized by a prolonged dormant season, resulting in pronounced seasonal imbalances in forage availability (2). Consequently, grazing Tibetan sheep often experience inadequate protein intake, particularly a deficiency in rumen-degradable protein, which limits their growth and development. To counteract these adverse effects, strategies such as grazing combined with supplementary feeding or fully confined feeding systems have been widely adopted. These approaches effectively alleviate issues such as weight loss and deterioration in body condition during the dormant forage season (3), particularly by ensuring adequate nitrogen intake to support animal growth performance. In recent years, the expanding scale of confined feeding for early-weaned lambs has steadily increased the demand for protein feed ingredients. In China, high-quality protein feed sources such as soybeans and soybean meal have long relied on imports. Their prices are therefore influenced by international market dynamics, limiting local control and severely impacting the profitability of livestock farming.

To reduce the reliance on high-quality feed ingredients such as soybean meal and lower costs, non-protein nitrogen (NPN) can provide rumen microorganisms with a nitrogen source, enabling the synthesis of microbial proteins at low cost (4). Urea, a key NPN source, offers high nitrogen content and is readily available, allowing it to replace conventional protein feeds in ruminant diets (5). Substituting a safe proportion of dietary soybean meal with urea not only reduces feed costs but also decreases nitrogen emissions while maintaining the animal growth performance (6). Numerous studies have shown that appropriate dietary NPN supplementation exerts no adverse effects on ruminant growth performance. Wahyono et al. (7) and Alizadeh et al. (8) reported that urea, as a low-cost nitrogen source, enhances rumen fermentation, nitrogen intake, and organic matter digestibility in sheep. Similarly, inclusion of moderate urea in Dorper crossbred sheep diets safely improved growth performance (9). Ma et al. (10) further found that slow-release urea promotes microbial protein synthesis, fiber digestion, and nitrogen retention. Collectively, these findings suggest that urea supplementation can enhance ruminant growth by modulating rumen fermentation and nitrogen metabolism. However, its effects on sheep meat flavor remain unclear.

Sheep meat flavor is strongly influenced by intramuscular fat and fatty acids composition (11), with adipose tissue serving as a metabolically active organ that plays an integral role in systemic metabolic regulation (12). Specifically, subcutaneous adipose tissue (SAT), the most abundant fat depot in ruminants, is closely linked to economically important traits and serves as a critical determinant of carcass and meat quality (13). The characteristic flavor of sheep meat, including its distinct meaty notes, largely arises from the metabolic transformation of lipids within adipose tissue (14), with fatty acid profiles conferring species-specific sensory characteristics (15). Fatty acids serve as the primary substrate for flavor development, and their structural diversity and relative abundance directly influence the resulting organoleptic properties (16). Sheep adipose tissue contains a complex mixture of saturated (SFA), monounsaturated (MUFA), polyunsaturated (PUFA), and branched-chain fatty acids (BCFA), each contributing uniquely to the overall flavor profile. The characteristic aroma of sheep meat primarily arises from the oxidative degradation of these fatty acids into volatile compounds. These trace-level constituents, primarily aldehydes, alcohols, ketones, and esters, with aldehydes being the key drivers of characteristic aroma, collectively define the unique meat flavor profile. Despite the established link between adipose tissue fatty acid composition and volatile compound formation, the impact of dietary urea supplementation on these parameters in subcutaneous fat remains largely unexplored. It is further hypothesized that excessive urea intake may exceed the ammonia utilization capacity of rumen microorganisms, potentially leading to systemic ammonia accumulation and subsequent deposition in adipose tissue, which could negatively affect meat flavor. Excess dietary urea undergoes rapid hydrolysis to ammonia. When ammonia production surpasses the combined capacities for microbial assimilation and hepatic detoxification, the excess enters the systemic circulation (17, 18). Owing to its high lipophilicity, ammonia readily diffuses into and accumulates within lipid-rich tissues such as SAT (19). However, this mechanism remains to be empirically validated.

In the current study, we hypothesized excess dietary urea causes ammonia accumulation in SAT, disrupting tissue morphology, lipid homeostasis, and flavor profiles. Therefore, this study aimed to investigate the effects of dietary urea supplementation on Tibetan sheep by examining adipose tissue morphology, fatty acid composition, lipidomic profiles, and volatile flavor compounds. The findings of this study will serve as a reference for the rational and efficient use of dietary urea in Tibetan sheep production, with implications for both growth performance and meat quality.

## 2 Materials and methods

### 2.1 Experimental design and feeding management

The study was conducted at the Jinzang Ecological Plateau Tibetan Sheep Breeding Professional Cooperative in Haiyan County, Haibei Prefecture, Qinghai Province. A total of 80 healthy, 2-month-old, early-weaned female Tibetan lambs (initial body weight: 16.2 ± 1.01 kg) were selected for this 105-day feeding trial, which included a 15-day adaptation period followed by a 90-day experimental phase. The lambs were randomly assigned to four dietary treatment groups (*n* = 20 per group), with four replicates per group and five lambs per replicate.

In accordance with the “Safety Specification for the Use of Feed Additives” promulgated by the Ministry of Agriculture and Rural Affairs of the PR China, the contribution of non-protein nitrogen to total dietary crude protein (CP) in total mixed rations should not exceed 30% (20). Based on this threshold, four urea levels were established: 0% (U0), 0.80% (U1), 1.19% (U2), and 1.61% (U3), corresponding to urea derived CP contributions of 0, 17, 26, and 35% of total dietary CP, respectively. This experimental design included two levels below the 30% safety limit, one level approaching the threshold, and one level exceeding it, thereby enabling evaluation of the physiological and metabolic responses to supra-threshold urea supplementation. In this study, we selected conventional feed-grade urea as NPN source with nitrogen content 46% (Beijing Kangpuhuiwei Technology Co., LTD, Beijing, China). The basal diet consisted of 70% concentrate and 30% roughage, with oat silage and oat hay provided at a 1:1 ratio on a dry matter basis. The details of dietary composition and nutrient profiles are provided in Table 1.

**Table 1:** Ingredient composition and nutritional levels (dry matter basis) of diets.

Items	Group			
	U0	U1	U2	U3
Ingredients/%				
Oat hay	15.0	15.0	15.0	15.0
Oat silage	15.0	15.0	15.0	15.0
Corn	38.3	39.3	46	52
Soybean meal	2.70	0.70	0.70	0.70
Canola seed meal	13.2	7.7	5.4	2.50
Cottonseed meal	3.11	1.40	1.40	0.98
Palm kernel meal	8.00	15.4	10.7	8.00
Urea	0	0.80	1.19	1.61
Salt	0.70	0.70	0.70	0.70
Baking soda	0.070	0.071	0.070	0.070
Limestone	0.70	0.70	0.70	0.70
4% Premix^1^	2.80	2.80	2.80	2.80
1% Premix^2^	0.42	0.42	0.42	0.42
Total	100	100	100	100
Nutritional level^3^				
DE, MJ/kg	11.3	11.0	11.2	11.2
EE, %	2.71	3.06	2.97	2.96
CP, %	13.2	13.3	13.3	13.3
NDF, %	31.5	35.2	31.9	29.8
ADF, %	19.0	20.1	18.4	17.2
Ash, %	4.9	4.6	4.3	4.0
Ca, %	0.84	0.83	0.82	0.80
P, %	0.220	0.220	0.230	0.240

1 Each kg of the 4% premix contained: CaHPO_4_ 85 g, Glucose 500 g, Maifanite 300 g, and Ammonium nicotinate.125 mg.

2 Each kg of the 1% premix contained: CuSO_4_ 1.5 g, MnSO_4_ 4 g, ZnO 5 g, Na_2_SeO 31 g, Ca (IO_3_)_2_ 1.5 g, CoSO_4_ 0.5 g, Vitamin A 145,000 IU, Vitamin D 334,000 IU, Vitamin E 250 mg, and Ethoxyquin 5 g.

3 Among the dietary nutrient components, crude protein (CP), ether extract (EE), neutral detergent fiber (NDF), acid detergent fiber (ADF), calcium (Ca), and total phosphorus (TP) were measured values, while digestible energy (DE) was a calculated value.

During the trial, Tibetan sheep were housed indoors and fed twice daily at 08:00 and 16:00, with ad libitum access to feed and water. Before the experiment, all animals were ear-tagged, and pens were thoroughly disinfected. Lambs were vaccinated before entry into the facility.

### 2.2 Sample collection

A 200 g representative feed sample was collected at the beginning of the experiment by the quartering method and ground to pass through a 1-mm screen for chemical analysis. Crude protein (CP) content was determined by the Kjeldahl method, and ether extract (EE) was measured by Soxhlet extraction. Neutral detergent fiber (NDF) and acid detergent fiber (ADF) were analyzed according to the procedures of Van Soest et al. (21). Calcium (Ca) and total phosphorus (P) concentrations were quantified by inductively coupled plasma optical emission spectrometry (ICP-OES; Spectro Arcos, SPECTRO Analytical Instruments, Kleve, Germany) following AOAC Official Method 968.08 (22).

At the end of the 105-day trial, five lambs were randomly selected from each treatment and slaughtered following a 24 h fast and 12 h water withdrawal. Immediately post-mortem, back SAT was collected. A portion of the tissue was rinsed with ice-cold physiological saline, trimmed into 1 × 1 × 1 cm^3^ cubes, and fixed in 4% paraformaldehyde for subsequent histological examination. The remaining tissue was snap-frozen in liquid nitrogen and stored at −80 °C for subsequent analysis of fatty acid composition, volatile flavor profiles, and lipidomic characterization.

### 2.3 Adipose tissue histomorphology analysis

SAT samples fixed in 4% paraformaldehyde were embedded in paraffin and sectioned at a thickness of 4 μm. Following deparaffinization and rehydration, sections were stained with hematoxylin and eosin and mounted with neutral balsam for microscopic examination, and mounted for morphological examination using an optical microscope (BX53, Olympus Corporation, Tokyo, Japan). The tissue sections were examined under an optical microscope, and images were captured at a magnification of 10×. ImageJ software (Version 2.13.0; National Institutes of Health, Bethesda, MD, United States) Adipocyte size was determined by measuring the longitudinal and transverse diameters of intact cells, with the mean values representing the cell diameter. Adipocyte density was calculated by determining the number of cells per unit area.

### 2.4 Determination of fatty acids in subcutaneous adipose tissue

Fatty acid composition was determined using the acid hydrolysis-extraction method in accordance with the National Food Safety Standard (GB 5009.168–2016) in China. SAT samples were subjected to direct saponification and transesterification to generate fatty acid methyl esters (FAMEs). The resulting organic phase was collected, dehydrated with anhydrous sodium sulfate, and filtered through a 0.22 μm organic membrane into an amber vial for subsequent analysis. Samples were analyzed in duplicate to ensure analytical precision, and any outliers were reanalyzed.

Fatty acid profiles were determined using a gas chromatograph (FL9720) equipped with a flame ionization detector and an SP-2560 capillary column (100 m × 0.25 mm × 0.20 μm). The heating temperature program was as follows: initial temperature of 100 °C for 5 min, followed by an increase of 4 °C/min to 240 °C, which was held for 30 min. The injector and detector temperatures were maintained at 260 °C. High-purity nitrogen served as the carrier gas, with a split ratio of 20:1 and an injection volume of 1.0 μL. FAMEs were identified by comparing their retention times with those of standard mixtures. Fatty acid quantification was performed based on the internal standard peak area method, and theoretical response factors were applied to correct for differences in detector sensitivity (23).

The nutritional quality of the fatty acid profile was assessed using the n-6/n-3 PUFA ratio, along with the indices of atherogenicity (AI) and thrombogenicity (TI). AI and TI were calculated as (24):


$$\begin{array}{l} \mathrm{AI}=\frac{[(C12:0)+(4\times C14:0)+(C16:0)]\;}{\left(\sum \text{MUFA}+\sum \text{PUFA}\right)};\\ \mathrm{TI}=\frac{\left[(C14:0)+(C16:0)+(C18:0)\right]}{\left[0.5\times \sum \text{MUFA}+0.5\times \sum n-6\;\text{PUFA}+3\times \sum n-3\;\text{PUFA}+(n-3)/(n-6)\right]}\; \end{array}$$

### 2.5 Lipid metabolism in subcutaneous adipose tissue

SAT samples stored at −80 °C were used to determine lipid using the method described by Chen (25). Briefly, 30 mg of lyophilized adipose tissue was powdered with pre-cooled PBS buffer and stainless-steel beads in a cryogenic grinder. Lipids were extracted twice using a chloroform-methanol mixture (2:1, v/v). The organic phases were pooled, washed with 0.9% (w/v) sodium chloride solution, dehydrated over anhydrous sodium sulfate, and filtered. The filtrate was evaporated to near dryness under a gentle stream of nitrogen and reconstituted in a methanol-acetonitrile mixture (1:1, v/v). The resulting solution was centrifuged at high speed under refrigerated conditions, and the supernatant was filtered through a 0.22 μm organic membrane for subsequent analysis.

Lipidomic profiling was conducted using an ultra-high-performance liquid chromatography-Q-Exactive Plus mass spectrometry system (Shimadzu, Kyoto, Japan; Thermo Fisher Scientific, Waltham, MA, United States). Chromatographic separation was achieved on a CSH C18 column (1.7 μm, 2.1 mm × 100 mm). The mobile phase consisted of a methanol–water solution (containing 5 mmol/L ammonium formate) and methanol-acetonitrile mixture, applied using a gradient elution program. Mass spectrometry data were acquired in both positive and negative electrospray ionization (ESI) modes (26), using full scan and multiple reaction monitoring (MRM) acquisition modes.

Lipid species were identified by matching retention times, exact molecular masses, and MS/MS fragmentation patterns against the Lipid Maps and HMDB databases. Absolute quantification of individual lipid species was performed using the isotope-labelled internal standards in combination with the external calibration curves. Multivariate analysis was conducted using orthogonal partial least squares discriminant analysis (OPLS-DA) using SIMCA software (version 14.1). Differential lipid species were screened based on variable importance in projection (VIP) values and Student’s t-test, with thresholds set at VIP > 1 and adjusted *p* < 0.05.

### 2.6 Flavor compounds in subcutaneous adipose tissue

Volatile flavor compounds were determined using headspace-gas chromatography-ion mobility spectrometry (HS-GC-IMS) (27). Briefly, 1.0 mL of SAT sample was placed into a 20 mL headspace vial, incubated for 15 min before being injected into the system. Each sample was analyzed in triplicate to ensure analytical reproducibility.

A standard mixture containing six ketones was used to establish a calibration curve between retention time and retention index (RI). Subsequently, the RI values of the target compounds were calculated based on their retention time. Qualitative analysis of the target compounds was performed by searching and matching against the GC retention index database (NIST 2020) and the IMS drift time database built into the VOCal software.

Data processing and visualization were conducted using VOCal software with integrated plugins, including Reporter, Gallery Plot, and Dynamic PCA. These tools were used to generate three-dimensional (3D) and two-dimensional (2D) spectra, difference spectra, fingerprint plots, and principal component analysis (PCA) score plots, enabling comprehensive comparison of volatile organic compounds (VOCs) among samples.

### 2.7 Statistical analysis

The density and diameter of subcutaneous adipocyte, along with fatty acid and flavor compound contents, were analyzed using one-way ANOVA in IBM SPSS 26 (IBM Corp., Armonk, NY, United States). Multiple comparisons among treatment groups were performed using Tukey’s test. *p* < 0.05 was considered statistically significant. Pearson correlation analysis was conducted to assess the relationships between key lipid molecules and volatile flavor compounds using cloud platform (Omicsmart, Gene Denovo, Guangzhou, China). Correlations were considered significant at *p* < 0.05 and |*r*| ≥ 0.80.

## 3 Results

### 3.1 Effects of dietary urea supplementation levels on SAT morphology in Tibetan sheep

**Figure 1:**
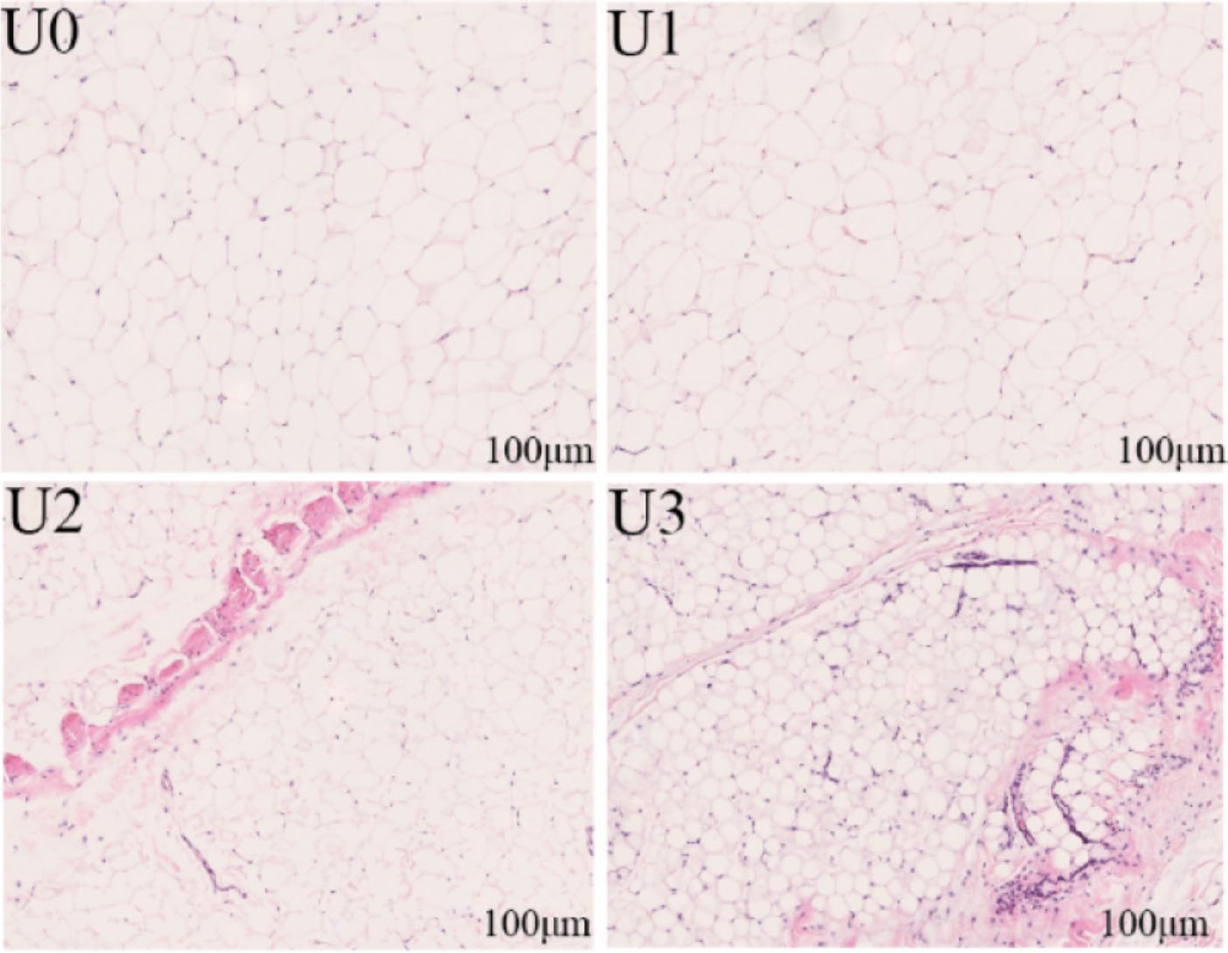
Influence of different dietary urea supplementation on the morphology of subcutaneous adipose tissue in Tibetan sheep. U0, control group; U1, 0.80% urea supplementation group; U2, 1.19% urea supplementation group; U3, 1.61% urea supplementation group.

Adipocyte diameter was significantly greater in the U0 group than in the U1 group (Table 2; *p* < 0.05). In both the U0 and U1 groups, adipocytes were densely packed, exhibiting distinct polygonal boundaries, uniform sizes, and intimate intercellular contacts. Increases in urea levels resulted in expansion of connective tissue within the SAT, accompanied by adipocyte rupture and diminished cellular compactness (Figure 1).

**Table 2:** Effects of different dietary urea levels on the morphology of subcutaneous adipose tissue in Tibetan sheep.

Items	Groups				SEM	*p-*value
	U0	U1	U2	U3		
Cell diameter/μm	100^a^	86^b^	93^ab^	88^ab^	1.96	0.048
Cell density (cells/field)	1,020	1,140	1,060	1,430	63	0.056

^a,b^Means within a row with different superscripts differ from each other (*p* < 0.05).

U0, control group; U1, 0.80% urea supplementation group; U2, 1.19% urea supplementation group; U3, 1.61% urea supplementation group.

### 3.2 Effects of dietary urea supplementation levels on fatty acid content in SAT of Tibetan sheep

As shown in Table 3, the contents of trans-fatty acids such as trans-9-hexadecenoic acid (C16:1 T), trans-11-octadecenoic acid (C18:1n11t), trans-7-nonadecenoic acid (C19:1n7t), and trans-9-nonadecenoic acid (C19:1n10t) were significantly higher in the U0 group than those in the U3 group (*p* < 0.05). Conversely, the content of cis-fatty acids such as oleic acid (C18:1n9c) was significantly higher in the U3 group compared to the U0 group (*p* < 0.05).

**Table 3:** Effects of different dietary urea levels on fatty acid content in subcutaneous adipose tissue of Tibetan sheep (mg/100 g).

Items	Groups				SEM	*p-*value
	U0	U1	U2	U3		
SFA						
C12:0	50	35.4	26.4	31.8	4.1	0.227
C13:0	2.10	2.05	1.69	1.49	0.148	0.45
C14:0	730	570	590	580	30.0	0.217
C15:0	59	63	57	49	3.71	0.61
C16:0	3,180	3,420	3,310	3,200	119	0.91
C17:0	142	161	158	120	11.5	0.64
C18:0	2,630	3,130	3,050	2,600	163	0.60
C20:0	26.2	21.6	22.2	22.7	1.65	0.81
C21:0	2.29	1.65	1.26	1.30	0.175	0.114
C22:0	3.41	2.72	2.40	2.86	0.250	0.61
C23:0	1.23	1.15	1.07	0.98	0.058	0.52
C24:0	1.13	0.97	0.72	0.74	0.070	0.076
MUFA						
C14:1	20.4	13.3	13.5	15.3	1.50	0.339
C15:1 T	7.9	6.6	7.7	6.0	1.10	0.94
C16:1 T	32.1^a^	24.0^ab^	20.9^ab^	17.4^b^	2.11	0.047
C16:1	332	361	346	379	15.4	0.783
C17:1 T	8.0	8.7	6.1	10.3	0.81	0.375
C17:1	69	81	79	72	4.8	0.85
C18:1n9t	155	115	133	76.3	11.5	0.062
C18:1n11t	57^a^	45^ab^	41^ab^	26.3^b^	4.2	0.045
C18:1n9c	1400^b^	2310^ab^	2690^a^	2810^a^	207	0.033
C18:1n11c	2,460	2,180	1810	1830	140	0.318
C19:1n7t	6.7^a^	3.74^b^	3.03^b^	3.43^b^	0.51	0.010
C19:1n10t	7.8^a^	5.6^ab^	4.1^bc^	3.08^c^	0.59	0.002
C20:1 T	0.87	0.97	1.00	0.82	0.059	0.74
C20:1	43^a^	16.3^b^	19.4^b^	19.7^b^	3.65	0.004
C22:1 T	1.20	1.17	1.24	1.07	0.063	0.85
C22:1	34.0^a^	10.7^b^	14.4^b^	11.4^b^	3.00	<0.001
PUFA						
C18:2n6c	215	190	189	143	14.5	0.40
C18:3n6	2.80	2.93	3.12	2.54	0.161	0.70
C18:3n3	29.7^a^	17.4^b^	17.9^b^	16.7^b^	1.94	0.016
C20:2	2.37^a^	1.41^b^	1.49^b^	1.26^b^	0.148	0.005
C20:3n6	2.71	2.75	2.78	2.37	0.130	0.71
C20:4n6	6.9	8.5	8.6	7.0	0.419	0.313
C22:2	1.33	0.99	0.94	0.88	0.068	0.050
C22:4	1.46	1.93	1.52	1.30	0.109	0.202
C22:5n6	6.3	5.8	5.9	5.7	0.194	0.77
Summary						
SFA	6,820	7,410	7,210	6,600	287	0.80
MUFA	4,640	5,180	5,190	5,280	191	0.70
PUFA	269	232	231	181	16.8	0.364
n-3PUFA	29.7^a^	17.4^b^	17.9^b^	16.7^b^	1.94	0.016
n-6PUFA	239	214	214	164	15.2	0.41
n-6/n-3PUFA	8.02^c^	12.4^a^	11.9^a^	9.8^b^	0.54	<0.001
AI^1^	2,960	2,330	2,390	2,350	123	0.209
TI^2^	212	162	163	135	12.7	0.187

1 AI, atherogenic index.

2 TI, thrombogenic index.

^a,b,c^Means within a row with different superscripts differ from each other (*p* < 0.05). SFA, Saturated fatty acid; MUFA, Monounsaturated fatty acid; PUFA, Polyunsaturated fatty acid U0, control group; U1, 0.80% urea supplementation group; U2, 1.19% urea supplementation group; U3, 1.61% urea supplementation group.

Furthermore, the contents of eicosenoic acid (C20:1), eicosadienoic acid (C20:2), *α*-linolenic acid (C18:3n3), and docosenoic acid (C22:1) were significantly higher in the U0 group than in the other three groups (*p* < 0.05). The content of n-3 polyunsaturated fatty acids (n-3 PUFA) was also significantly higher in the U0 group compared to the other three groups (*p* < 0.05). The n-6/n-3 PUFA ratio in the U1 and U2 groups was significantly higher than in the U0 and U3 groups (*p* < 0.01).

### 3.3 Effects of dietary urea supplementation levels on the SAT lipidome in Tibetan sheep

**Figure 2:**
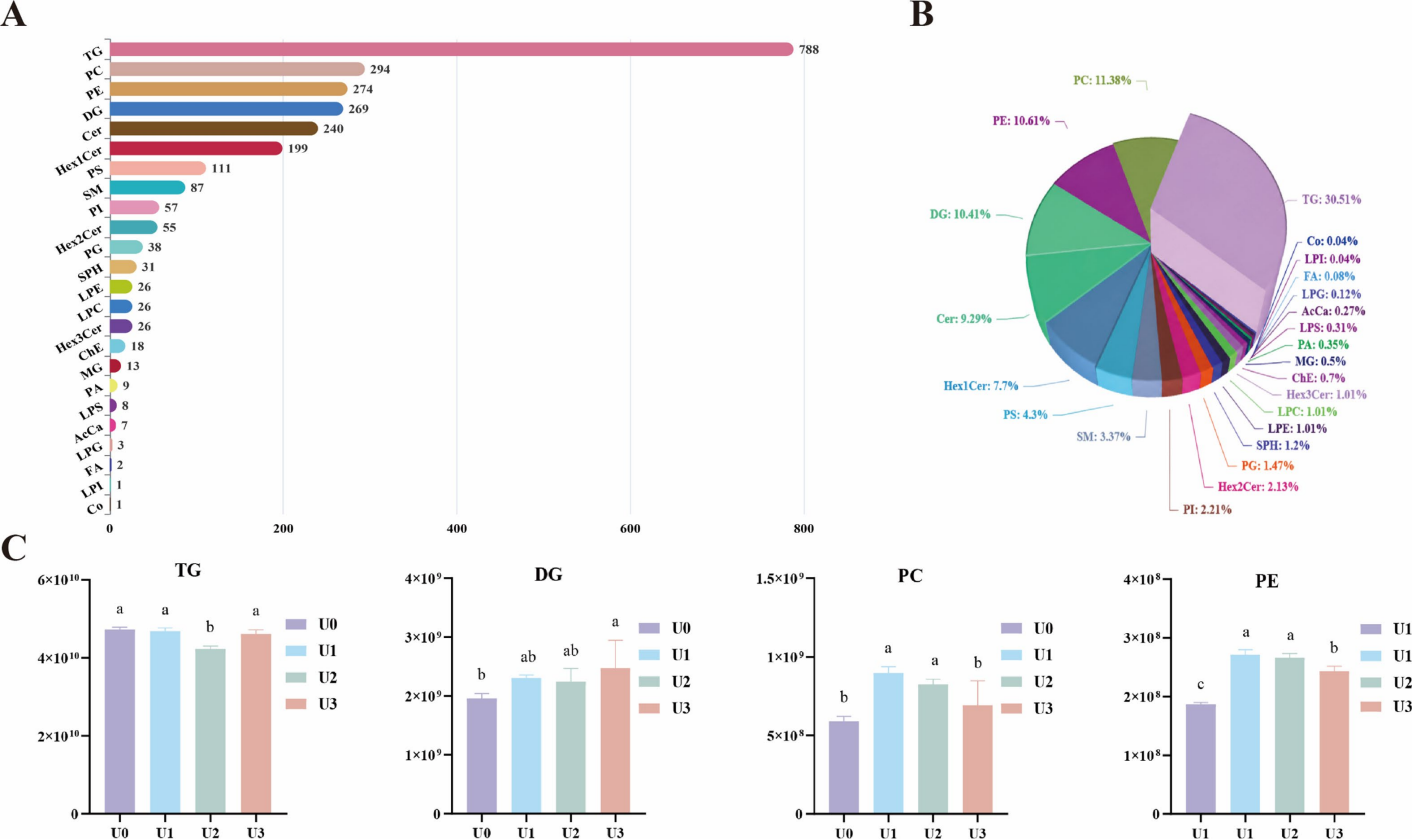
Lipidomic characteristics of subcutaneous fat and changes in key lipid molecule contents under different dietary urea levels in Tibetan sheep. **(A)** Horizontal bar chart showing the number of identified lipid molecules belonging to each lipid class. **(B)** Pie chart illustrating the relative proportion of each lipid class based on total lipid abundance. **(C)** Bar graphs displaying the relative contents of four major lipid subclasses (TG, DG, PC, PE) in subcutaneous fat of Tibetan sheep under four dietary urea treatments (U0–U3). **(A–C)** Means within a row with different superscripts differ from each other (*p* < 0.05). U0, control group; U1, 0.80% urea supplementation group; U2, 1.19% urea supplementation group; U3, 1.61% urea supplementation group. TG, Triacylglycerol; PC, Phosphatidylcholine; PE, Phosphatidylethanolamine; DG, Diacylglycerol; PS, Phosphatidylserine; PI, Phosphatidylinositol; PG, Phosphatidylglycerol; PA, Phosphatidic acid; LPC, Lysophosphatidylcholine; LPE, Lysophosphatidylethanolamine; LPS, Lysophosphatidylserine; LPG, Lysophosphatidylglycerol; LPI, Lysophosphatidylinositol; Cer, Ceramide; Hex1Cer, Monohexosylceramide; SM, Sphingomyelin; Hex2Cer, Dihexosylceramide; SPH, Sphingosine; Hex3Cer, Trihexosylceramide; AcCa, Acylcarnitine; ChE, Cholesteryl Ester; MG, Monoacylglycerol; FA, Fatty Acid; Co, Cholesterol.

High-throughput LC–MS analysis in both positive and negative ion modes identified a total of 2,583 lipid molecules (Figure 2A), among which 24 distinct lipid metabolites were annotated. Triglycerides (TG), phosphatidylcholine (PC), phosphatidylethanolamine (PE), and diglycerides (DG) were the predominant lipid classes (Figure 2B), accounting for 30.51, 11.38, 10.61, and 10.41% of the total lipid pool, respectively. As shown in Figure 2C, the TG content in the U2 group was significantly lower than that in the other three groups (*p* < 0.05), while the DG content in the U3 group was significantly higher than that in the U0 group (*p* < 0.05). Additionally, the contents of PC and PE in the U1 and U2 groups were significantly higher than those in the U0 and U3 groups (*p* < 0.05).

**Figure 3:**
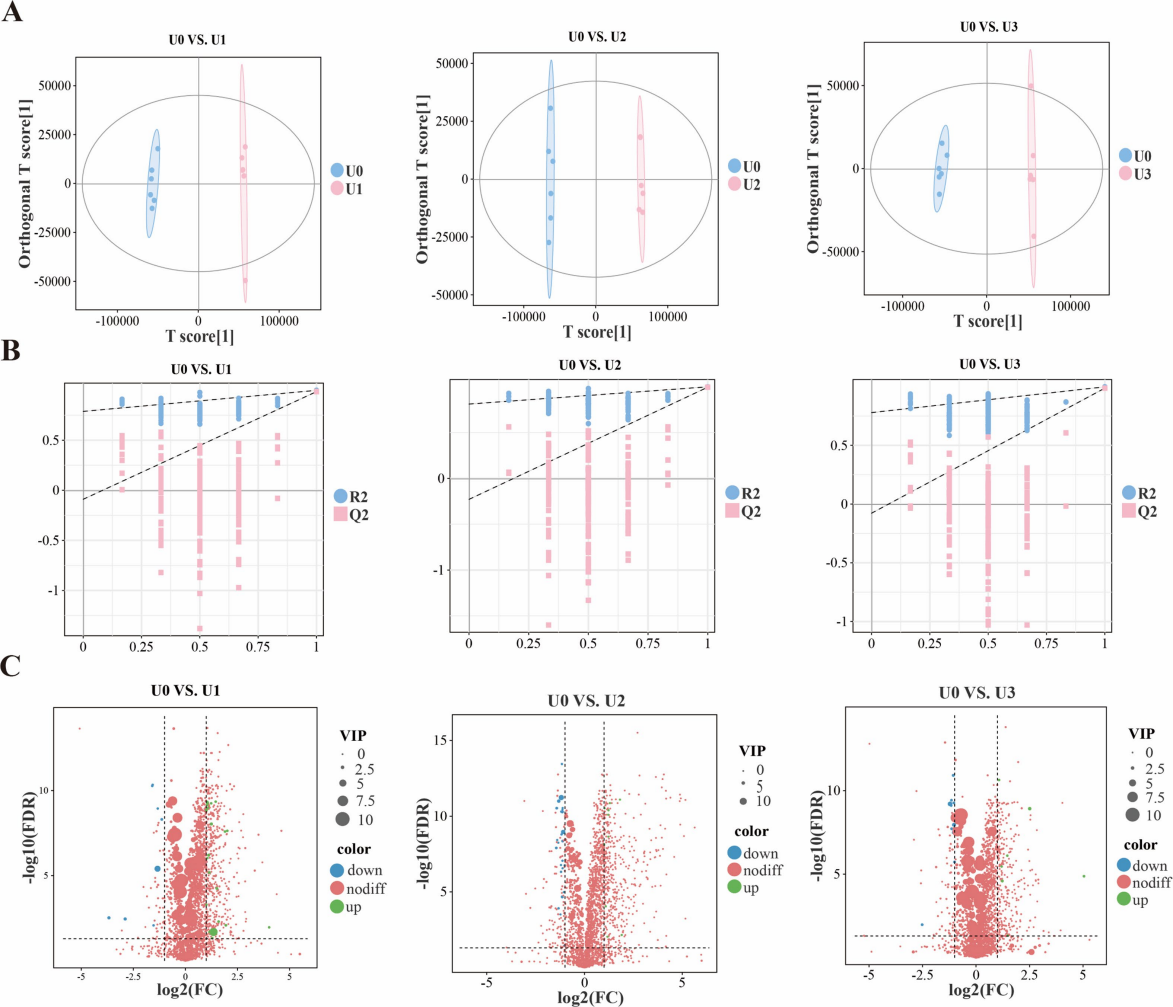
Orthogonal partial least squares discrimination analysis (OPLS-DA) and differential lipids filtration in subcutaneous adipose of Tibetan sheep. **(A)** Score plot of OPLS-DA analysis. **(B)** Permutation test of OPLS-DA. **(C)** Volcano plot of the differential lipids. Lipids with |*log2Fold change*| ≥ 2, adjusted *p* < 0.05 regarded as differential lipids. U0, control group; U1, 0.80% urea supplementation group; U2, 1.19% urea supplementation group; U3, 1.61% urea supplementation group.

Orthogonal partial least squares discriminant analysis (OPLS-DA) showed distinct separation among the treatment groups (Figure 3A). Permutation tests of the PLS-DA indicated that, for the U0 vs. U1, U0 vs. U2, and U0 vs. U3 comparisons, the R^2^X, R^2^Y, and *Q*^2^ values were 0.00, 0.79, and −0.09; 0.00, 0.81, and −0.23; 0.00, 0.78, and −0.08, respectively (Figure 3B). The R^2^Y and Q^2^ values indicated that the models were robust and not overfitted, supporting their suitability for subsequent screening of differential lipids.

As illustrated in Figures 3C, a total of 48 differential lipid species were identified. In the U0 vs. U1 comparison, 22 differential lipid molecules were detected, of which 17 were upregulated and 5 were downregulated. In the U0 vs. U2 comparison, 22 differential lipid molecules were detected, including 8 upregulated and 14 downregulated. In the U0 vs. U3 comparison, 4 differential lipids were detected, with 3 upregulated and 1 downregulated.

**Figure 4:**
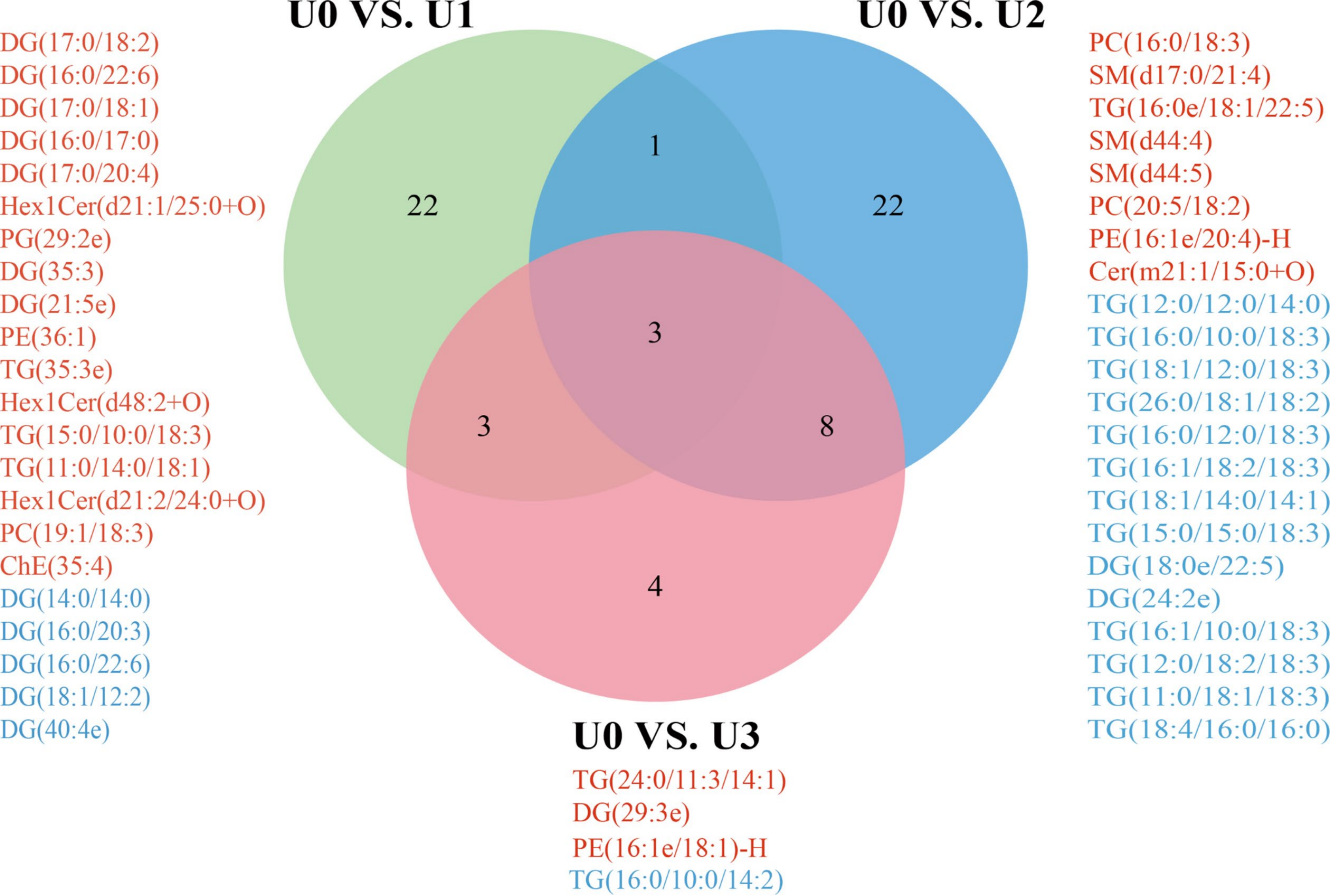
Venn diagram of differential lipids (red: Upregulated; blue: Downregulated). Venn diagram illustrating the distribution and overlap of differential lipid molecules among the three comparison groups (U0 vs. U1, U0 vs. U2, and U0 vs. U3). The numbers within each region indicate the count of lipid molecules.

As shown in Figure 4, using the thresholds of |log₂ Fold change| ≥ 2, and adjusted *p* < 0.05, 3 differential lipids were common to the U0 vs. U1, U0 vs. U2, and U0 vs. U3 comparisons. In addition, 22 unique differential lipids were found in the U0 vs. U1 comparison, 22 unique lipid molecules in the U0 vs. U2 comparison, and 4 unique lipid molecules in the U0 vs. U3 comparison.

### 3.4 Effects of dietary urea supplementation levels on volatile flavor compounds in SAT of Tibetan sheep

**Figure 5:**
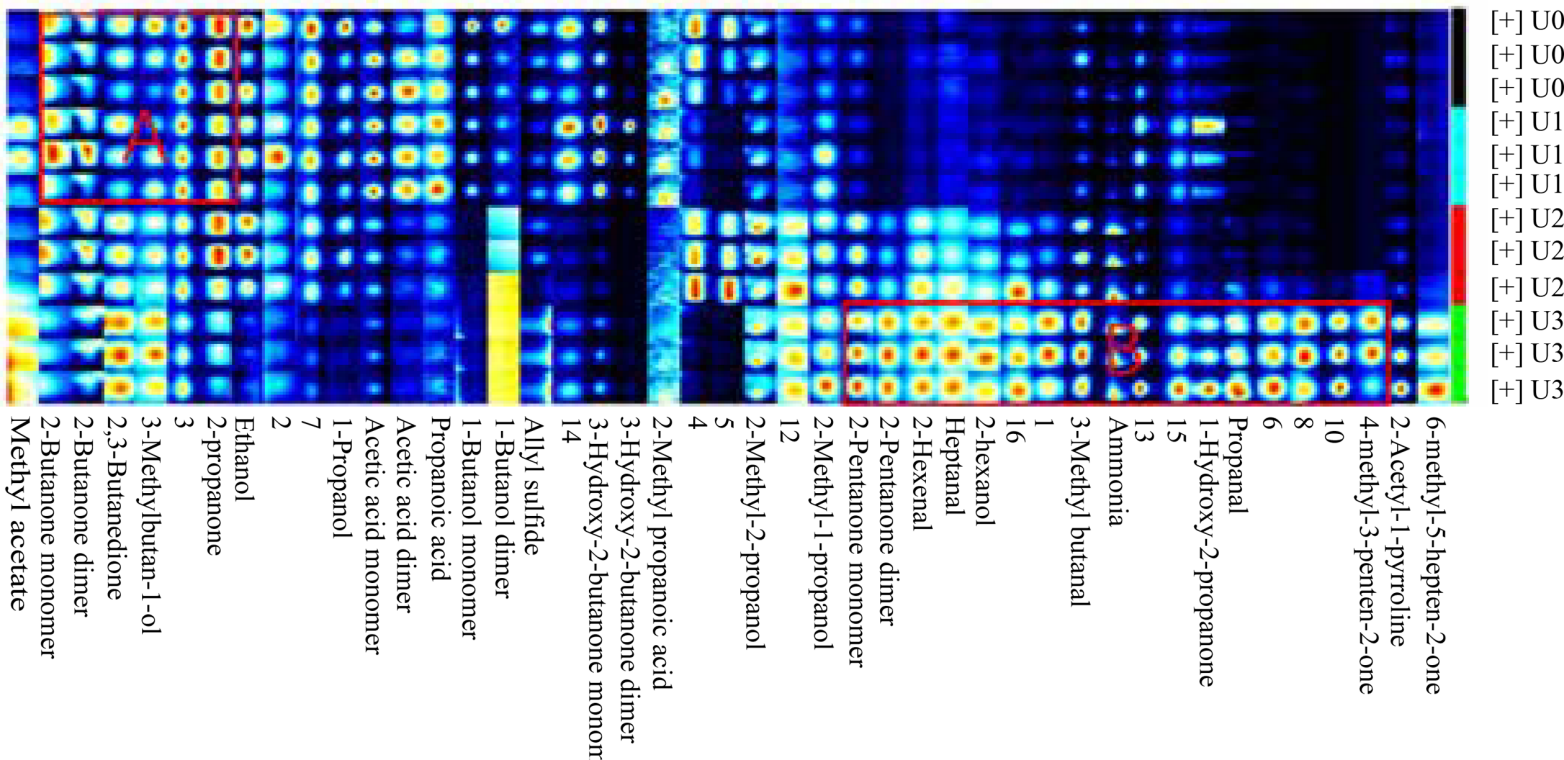
Fingerprint chromatogram of volatile flavor compounds in the subcutaneous adipose of Tibetan sheep, where each row = one sample (U0–U3); each column = one volatile compound. Color gradient (blue → red → white) indicates peak intensity (low → high). Red boxes mark characteristic regions: A = U0/U1-enriched compounds; B = U3-specific compounds. U0, Control group; u1, 0.80% urea supplementation group; u2, 1.19% urea supplementation group; u3, 1.61% urea supplementation group.

Figure 5 illustrates the volatile organic compound (VOC) profiles across dietary treatments. The U0, U1, and U2 groups displayed similar VOC profiles, whereas the U3 group showed a markedly distinct composition. Specifically, Region A represents VOCs with higher signal intensities in the U0, U1, and U2 groups, primarily including 2-butanone (monomer and dimer), acetone, and ethanol. In contrast, Region B highlights compounds more enriched in the U3 group, such as ammonia, 2-hexanol, heptanal, and 2-pentanone (monomer and dimer).

As shown in Table 4, dietary urea supplementation significantly altered the volatile profile of SAT in Tibetan sheep (*p* < 0.05). Ammonia concentration increased proportionally with dietary urea inclusion (*p* < 0.05), contributing to an intensified pungent odor in the adipose tissue. Furthermore, the concentration of 2-acetyl-1-pyrroline was significantly higher in the U3 group compared to the other treatments (*p* < 0.05). Conversely, the levels of characteristic flavor-active compounds, such as alcohols and acids (e.g., ethanol and acetic acid) decreased significantly as urea levels increased (*p* < 0.05), potentially diminishing the intensity of alcoholic and acidic notes. Additionally, buttery-flavored compounds, such as 3-hydroxy-2-butanone dimer, were significantly more abundant in the U0 and U1 groups than in the U2 and U3 groups (*p* < 0.05).

**Table 4:** Effect of dietary urea levels on volatile flavor compounds in subcutaneous adipose tissue of Tibetan sheep (%).

Items	Flavor	Groups				SEM	*p-*value
		U1	U2	U3	U4		
Ammonia	Ammoniacal	29.3^b^	33.5^b^	64^a^	60^a^	4.9	<0.001
2-Propanone	Spicy, caramel-like, fresh	15.4^a^	12.0^a^	7.7^b^	3.64^c^	1.38	<0.001
Ethanol	Pungent, alcoholic	7.0^a^	5.4^ab^	4.0^ab^	1.34^b^	0.74	0.014
Acetic acid monomer	Acidic	6.2^a^	6.0^a^	2.06^b^	1.24^b^	0.69	<0.001
3-Hydroxy-2-butanone monomer	Buttery, creamy	4.9^a^	6.5^a^	0.76^b^	1.28^b^	0.74	<0.001
2-Butanone dimer	Fruity, camphoraceous	2.46^a^	2.56^a^	1.55^ab^	1.08^b^	0.220	0.013
2-Butanone monomer	Fruity, camphoraceous	2.37^a^	2.45^a^	1.08^b^	0.57^b^	0.255	<0.001
3-Methyl butanal	Alcoholic, fruity	1.67^ab^	1.16^b^	1.52^ab^	2.37^a^	0.166	0.034
2-Methyl-2-propanol	Camphoraceous	1.54	1.27	1.34	1.09	0.074	0.192
1-Butanol monomer	Alcoholic (whiskey), fruity (banana)	1.40^a^	1.06^a^	0.203^b^	0.370^b^	0.161	0.001
3-Hydroxy-2-butanone dimer	Buttery, creamy	1.27^ab^	3.16^a^	0.204^b^	0.290^b^	0.420	0.012
1-Propanol	Fresh, alcoholic, leathery	1.23^a^	0.99^ab^	0.55^bc^	0.180^c^	0.135	0.003
2-Acetyl-1-pyrroline	Minty	1.02^b^	0.66^c^	0.55^c^	1.36^a^	0.101	<0.001
1-Hydroxy-2-propanone	Buttery, vanilla, spicy, malty	0.44^ab^	1.04^a^	0.110^b^	0.49^ab^	0.117	0.009

^a,b,c^Means within a row with different superscripts differ from each other (*p* < 0.05).

U0, control group; U1, 0.80% urea supplementation group; U2, 1.19% urea supplementation group; U3, 1.61% urea supplementation group.

### 3.5 Correlation analysis between key lipid molecules and flavor compounds in SAT

Differential lipid molecules were screened using the thresholds of |log₂ Fold change| ≥ 2 and adjusted *p* < 0.05. A total of 23 differential lipid molecules were identified, including 3 shared among all three comparisons, 12 unique to the U1 vs. U0 comparison, 6 unique to the U2 vs. U0 comparison, and 3 unique to the U3 vs. U0 comparison. Pearson correlation analysis was further performed between these 23 differential lipids and key volatile flavor compounds.

**Figure 6:**
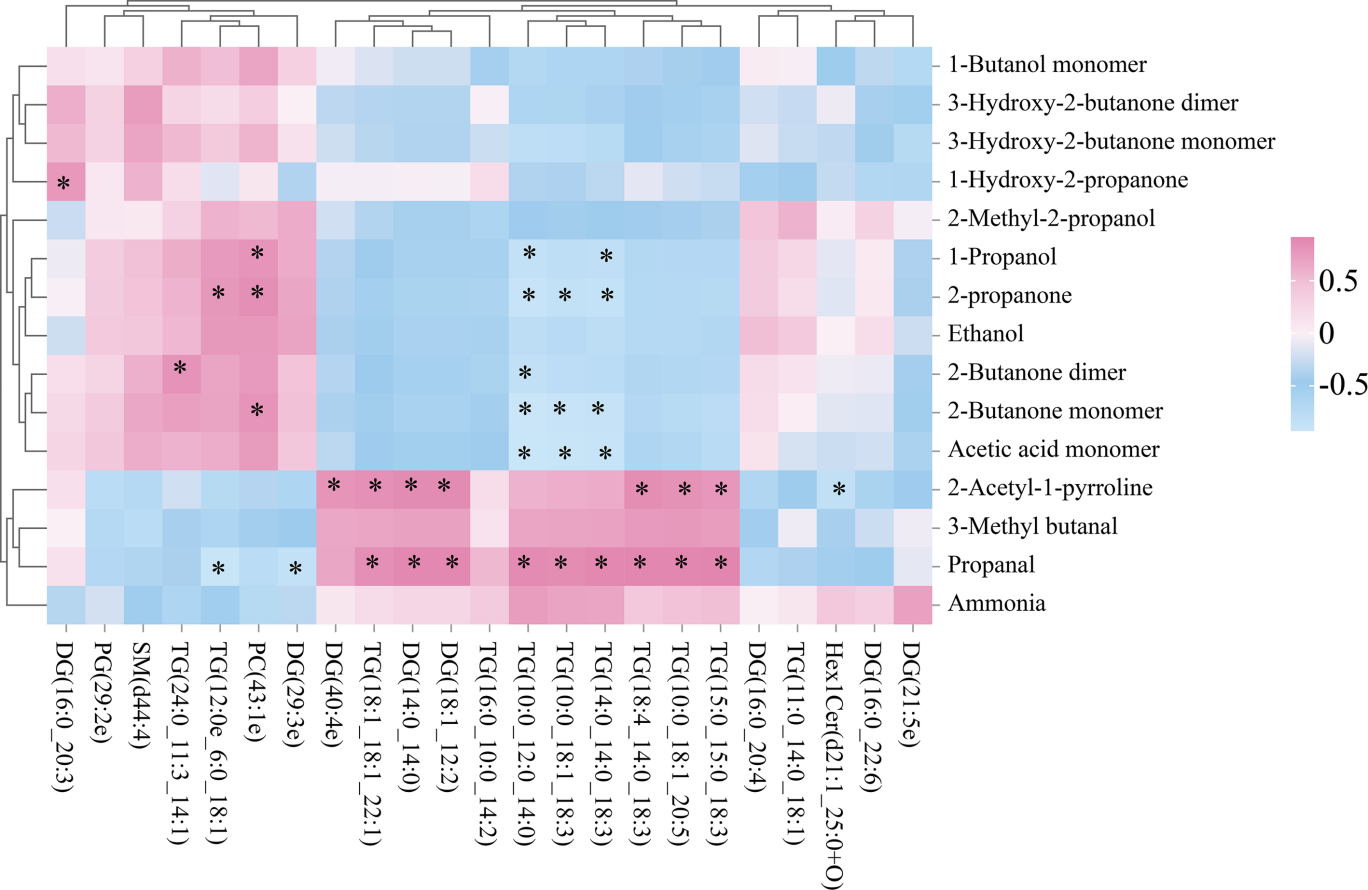
Correlation heatmap between key volatile flavor compounds and differential lipid molecules in subcutaneous adipose of Tibetan sheep. *Indicates significant correlation (|*r*| ≥ 0.80, *p* < 0.001).

As shown in Figure 6, TG (24:0_11:3_14:1) was significantly positively correlated with 2-butanone dimer (*r* = 0.83, *p* < 0.001). TG (12:0e_6:0_18:1) was significantly positively correlated with acetone (*r* = 0.80, *p* < 0.001). TG (18:1_18:1_22:1) was significantly positively correlated with 2-acetyl-1-pyrroline and propanal (*r* = 0.86, *p* < 0.001). TG (10:0_12:0_14:0) was significantly positively correlated with propanal (*r* = 0.89, *p* < 0.001). TG (10:0_18:1_18:3) was significantly positively correlated with propanal (*r* = 0.88, *p* < 0.001). TG (14:0_14:0_18:3) was positively correlated with propanal (*r* = 0.91, *p* < 0.001). TG (18:4_14:0_18:3), TG (10:0_18:1_20:5), and TG (15:0_15:0_18:3) were significantly positively correlated with 2-acetyl-1-pyrroline and propanal (*r* = 0.80 ~ 0.93, *p* < 0.001). Diglyceride DG (16:0_20:3) was positively correlated with 1-hydroxy-2-propanone (*r* = 0.80, *p* < 0.001). DG (40:4e) was positively correlated with 2-acetyl-1-pyrroline (*r* = 0.82, *p* < 0.001). DG (14:0_14:0) and DG (18:1_12:2) were positively correlated with 2-acetyl-1-pyrroline and propanal (*r* = 0.85 ~ 0.91, *p* < 0.001). Phosphatidylcholine PC (43:1e) was significantly positively correlated with 2-butanone monomer, acetone, and 1-propanol (*r* = 0.83 ~ 0.86, *p* < 0.001).

TG (12:0e_6:0_18:1) was negatively correlated with propanal (*r* = −0.89, *p* < 0.001). TG (10:0_12:0_14:0), TG (10:0_18:1_18:3), and TG (14:0_14:0_18:3) were significantly negatively correlated with, 1-propanol, acetone, 2-butanone monomer, and acetic acid monomer (*r* = −0.82 ~ −0.92, *p* < 0.001). DG (29:3e) was significantly negatively correlated with propanal (*r* = −0.84, *p* < 0.001).

## 4 Discussion

Adipose tissue serves as the primary energy reservoir in animals, mobilizing fatty acids through lipolysis to meet physiological demands during fasting, stress, or lactation (28). The deposition of subcutaneous and visceral fat is a critical determinant of carcass quality and dressing percentage, with these processes being intrinsically linked to adipocyte morphology and function (29). Adipocyte diameter serves as a reliable indicator of lipid storage capacity, with a reduction in cell size typically signifying diminished intracellular lipid accumulation (30). In the present study, dietary urea supplementation consistently reduced adipocyte diameter compared with the control (U0). The significant decrease observed in the U1 group suggests that moderate urea inclusion effectively mitigates adipocyte hypertrophy. Although the reduction in the U2 and U3 groups did not reach statistical significance, the persistent downward trend across all urea-supplemented groups indicates a dose-dependent inhibition of lipid storage.

Increased adipocyte density is commonly interpreted as a compensatory response, whereby enhanced proliferation or differentiation of pre-adipocytes offsets a reduction in individual cell volume to maintain overall adipose tissue mass (31). In this study, adipocyte density increased with rising urea levels, peaking in the U3 group at approximately 40.6% above the control. Notably, cellular rupture was observed in the U2 and U3 groups, indicating potential structural impairment. This morphological disruption may be attributed to excessive ammonia intake, which can induce intracellular ammonia accumulation, disrupt pH homeostasis, and trigger oxidative stress, thereby compromising membrane integrity (32). While these changes in density were not statistically significant, the observed morphological disarray suggests that a dietary urea concentration of 1.19% (U2) represents a critical threshold for adipose tissue structural integrity. Further research is needed to elucidate the specific molecular mechanisms underlying this urea-induced morphological disruption. Fatty acids (FA) serve as primary flavor precursors in meat, influencing both intramuscular fat deposition and the release of volatile compounds through lipid oxidation (33). FA composition significantly dictates the textural properties of adipose tissue, particularly in subcutaneous and intermuscular fat depots. Although the rapid oxidation of unsaturated fatty acids (UFA), especially PUFA, is a critical factor for meat shelf-life and color stability, it is also essential for developing characteristic aromas during thermal processing (34). In this study, dietary urea supplementation exerted a concentration-dependent effect on the FA profile of SAT in Tibetan sheep, likely by modulating rumen microbial ecology, *de novo* FA synthesis, and biohydrogenation pathways. Trans-fatty acids (TFAs) are primarily derived from the isomerization of UFA (35), and their excessive accumulation can compromise cell membrane fluidity and lipid metabolic homeostasis (36). We observed a concentration-dependent decrease in trans-MUFA, specifically C16:1 T and C18:1n9t, in urea-supplemented groups compared with the control. Rumen biohydrogenation is primarily mediated by microbial taxa such as *Butyrivibrio* and *Ruminococcus*, which play key roles in the formation of TFAs (37, 38). Urea hydrolysis generates a substantial amount of ammonia nitrogen (NH_3_-N), which may elevate ruminal pH. This shift in pH can inhibit the activity of lipases and isomerases, thereby attenuating biohydrogenation efficiency (39) and limiting the conversion of cis-FA to trans isomers. This interpretation is supported by the observed increase in the concentration of oleic acid (C18:1n9c) in the U3 group compared with the U0 group. These findings suggest that dietary urea supplementation may inhibit biohydrogenation and TFA synthesis while promoting the accumulation of cis-monounsaturated FA, such as oleic acid. This shift could potentially enhance the nutritional quality of the lipid system (40), a result consistent with previous reports indicating that high NPN diets reduce beneficial FA such as conjugated linoleic acid (CLA) (41).

Regarding PUFA, the significantly higher concentrations of *α*-linolenic acid (C18:3n3) and total n-3 PUFA in the U0 group suggest that dietary urea supplementation may interfere with long-chain FA synthesis or PUFA preservation in adipocytes. As essential fatty acids, n-3 PUFAs play a critical role in human cardiovascular health (42, 43), and their accumulation in adipose tissue is largely dependent on the bypass efficiency of dietary lipids in the rumen. Excessive dietary urea may alter fermentation patterns, thereby inhibiting fatty acid synthase (FASN), the rate-limiting enzyme for *de novo* long-chain FA synthesis (44), potentially reducing the synthesis of long-chain FAs. Although total n-6 PUFA content did not differ significantly among treatments, the n-6/n-3 ratio was notably higher in the U1 and U2 groups. An elevated n-6/n-3 ratio is known to be associated with pro-inflammatory states and increased risk of atherosclerosis (45). Maintaining this ratio within recommended limits is vital for ensuring the nutritional quality of meat. Furthermore, although the reductions in the AI and TI following urea supplementation were not statistically significant, the observed downward trend suggests that moderate urea inclusion may contribute to a more favorable cardiovascular risk profile in mutton. In conclusion, dietary urea levels differentially regulate the FA composition of SAT in Tibetan sheep. While the urea concentration in the U3 group effectively reduced TFA content, it simultaneously compromised the accumulation of beneficial n-3 PUFAs. Conversely, the U0 group exhibited higher n-3 PUFA levels along with greater TFA content. Therefore, optimized urea supplementation (e.g., U2) may represent a strategic balance by reducing potentially harmful FAs while maintaining the proportion of beneficial fatty acids, thereby promoting a favorable FA profile and mitigating cardiovascular health risks.

Lipids are the primary energy reservoir in animals and play a crucial role in maintaining physiological homeostasis (46). The lipidome—comprising glycerides, phospholipids, free FA, and cholesterol esters—directly modulates fat deposition efficiency, energy metabolism, fatty acid composition, and the oxidative stability of meat, all of which are critical to the sensory and nutritional quality of mutton. In this study, a total of 2,583 lipid molecules were identified in SAT, with TG, PC, PE, and DG being the predominant classes. TG, an important contributor to the characteristic ‘mutton aroma’ (47), was significantly lower in concentrations in the U2 group compared with the other treatments. This reduction may be linked to the metabolic shifts induced by moderate urea supplementation. Previous studies have demonstrated that urea can upregulate the expression of genes associated with gluconeogenesis and energy metabolism (48). We hypothesize that moderate dietary urea supplementation enhances propionate production, thereby favoring hepatic gluconeogenesis and shifting carbon flux toward glycogen synthesis at the expense of lipogenesis. Phosphatidic acid (PA) can be enzymatically converted into DG, which are subsequently incorporated into TG for storage in lipid droplets (49). In this study, DG content was significantly higher in the U3 group compared to the U0 group and increased with rising dietary urea concentrations, suggesting that urea promotes this conversion. PC and PE, key constituents of cellular membranes, are vital for maintaining adipocyte structural integrity and metabolic activity, which in turn influence the formation of volatile flavor compounds (50). PC and PE levels were significantly higher in the U1 and U2 groups than in the U0 and U3 groups, aligning with previous findings (51) that moderate ammonia nitrogen supply optimizes phosphodiesterase activity and PC synthase expression. Conversely, excessive urea intake likely inhibits these enzymatic activities, disrupting membrane phospholipid homeostasis and contributing to the reduced adipocyte size observed in the high-urea concentration groups. In conclusion, dietary urea supplementation exerts a regulatory effect on the lipidomic profile of SAT in Tibetan sheep, specifically modulating the levels of TG, PC, PE, and DG. These shifts in lipid composition reflect broader alterations in energy metabolism and adipocyte homeostasis. Our findings suggest that moderate dietary urea supplementation (U1 and U2) supports metabolic stability, whereas excessive urea intake (U3) disrupts normal lipid turnover and adipocyte function, ultimately compromising the flavor precursors and reducing nutritional quality of the meat. The aroma profile of mutton is largely determined by the concentration and composition of volatile organic compounds (VOCs) (52). In this study, HS-GC-IMS analysis revealed that dietary urea supplementation significantly modulated the VOC profile in the SAT of Tibetan sheep. The primary volatile constituents identified were aldehydes, alcohols, ketones, and ammonia, with their relative abundances varying in a urea concentration-dependent manner. Alcohols, which largely originate from lipid oxidation and Strecker degradation, typically have high sensory thresholds, thereby exerting a limited impact on meat aroma compared with other volatile classes (53). We observed significantly higher concentrations of ethanol, butanol, and propanol in the control (U0) and low-dose (U1) groups compared with the medium- and high-dose (U2 and U3) groups. This pattern suggests that elevated ruminal ammonia levels under high urea intake may alter microbial metabolic pathways, potentially favoring ammonia utilization over the oxidation of electron donors such as alcohols (54). As a result, alcohol accumulation is reduced, likely diminishing the characteristic acidic and refreshing sensory notes in the final product. Ketones, which are derived from fatty acid oxidation, amino acid degradation, and microbial metabolism, are critical contributors to the characteristic buttery, creamy, and fruity notes of mutton. Our results demonstrate that the concentrations of key ketones—including acetone, 3-hydroxy-2-butanone, and 2-butanone dimers—were significantly higher in the U0 and U1 groups compared to the U2 and U3 groups. This suggests that low-concentration dietary urea supplementation may optimize the accumulation of these desirable flavor precursors. In contrast, high-concentration dietary urea appears to inhibit the accumulation of these compounds while simultaneously increasing ammonia concentration in the adipose tissue. The elevated ammonia in the U2 and U3 groups likely contributes to the pungent off-odors, thereby significantly compromising the overall sensory quality of the meat.

Lipids play a pivotal role in meat flavor development, serving both as solvents for flavor compounds and as essential flavor precursors. Through lipolysis and oxidation, lipids produce various flavor precursors that subsequently influence meat flavor quality (55). Correlation analysis between differential lipid molecules and volatile flavor compounds revealed that variations in subcutaneous fat lipids, particularly TG and DG, were significantly associated with several key volatile flavor compounds, such as aldehydes, ketones, and alcohols. Previous research has demonstrated that neutral lipids TG and phospholipids are crucial for flavor retention in meat and meat products (56). Most TGs (e.g., 24:0_11:3_14:1, 12:0e_6:0_18:1) showed significant positive correlation with various ketones, including 2-butanone dimer, acetone. This finding is consistent with Ma et al. (57), who reported a significant positive correlation between TG (16:0) and acetone following dietary supplementation with palm kernel meal. Ketones primarily originate through the oxidative degradation of unsaturated fatty acids (UFAs) and Maillard reactions, imparting characteristic milky and mildly sweet aromas. As the primary energy storage form in subcutaneous fat, TGs with higher UFAs are more susceptible to oxidation, thereby promoting ketone formation and contributing to the distinctive flavor profile of mutton. Several TGs, including (12:0e_6:0_18:1), (10:0_12:0_14:0), (18:4_14:0_18:3), and (10:0_18:1_20:5), were significantly positively correlated with propanal. Aldehydes are derived from the decomposition of UFAs, with a smaller portion originating from the Maillard reaction of sugars (58). Straight-chain aldehydes, alcohols, hydrocarbons, and ketones, among the volatile flavor compounds in meat products, are primarily produced through the hydrolysis and oxidation of TGs and phospholipids (59). At low concentrations, propanal imparts a light grassy aroma to Tibetan mutton, enriching its flavor profile. However, excessive TG oxidation and the subsequent accumulation of propionaldehyde result in rancidity and off-flavors, thereby leading compromising overall quality (60). These findings suggest that the observed correlations between TGs and propanal provide an important theoretical basis for understanding the regulatory mechanisms of urea supplementation on lipid oxidation and flavor quality of SAT in Tibetan sheep. Additionally, certain TGs, such as 10:0_12:0_14:0, 10:0_18:1_18:3, and 14:0_14:0_18:3, showed negative correlations with 1-propanol, acetone, 2-butanone dimer, and 2-butanone monomer.

This may be explained by the role of TGs as precursors, which undergo oxidative decomposition to generate flavor compounds such as ketones and alcohols, while metabolic regulation simultaneously inhibits their synthesis, ultimately resulting in a significant negative correlation in their abundance. In summary, the observed significant correlations between differential lipid molecules and volatile flavor compounds underscore the central regulatory role of lipid metabolism in meat flavor. Future research could further integrate analysis of key metabolic enzymes (e.g., lipoxygenase, acetyl-CoA, etc.) with gene expression profiling to deeply verify the metabolic pathways underlying lipid transformation and flavor development. Such approaches would provide an important basis for elucidating the mechanisms by which urea supplementation regulates the flavor of subcutaneous fat in Tibetan sheep.

Dietary urea supplementation significantly affected mutton flavor quality through modulation of lipid metabolism, and the volatile profile of subcutaneous fat. From a practical perspective, the cost savings achieved by replacing expensive protein feeds with urea must be balanced against the risk of flavor impairment. Supplementation at 0.80% urea (U1) provided an optimal compromise: feed costs were reduced while desirable buttery and creamy aroma compounds were preserved or increased, without generating ammonia-like off-odors that reduce sensory acceptability. In contrast, higher inclusion levels (U2 and U3) further decreased feed costs but led to marked flavor deterioration, characterized by reduced levels of favorable precursors and elevated pungent off-odors. Thus, urea supplementation at 0.80% offers a practical means of lowering production costs while maintaining the sensory quality of Tibetan sheep meat.

## 5 Conclusion

In conclusion, dietary urea supplementation modulates lipid metabolism and volatile flavor profiles in the SAT of Tibetan sheep in a dose-dependent manner. While urea effectively reduces trans-fatty acid deposition and alters the composition of key glycerolipids and glycerophospholipids, moderate inclusion (0.80%) promotes desirable milky and vanilla aromas. In contrast, urea inclusion at or above 1.19% compromises adipocyte structural integrity and induces lipid metabolic dysfunction. Notably, at the highest level (1.61%), accumulation of unassimilated ammonia in adipose tissue generates pungent off-odors and strongly inhibits the formation of favorable volatile compounds (buttery, creamy, and fruity notes), thereby severely impairing meat flavor. These findings indicate that although urea is a viable and cost-effective nitrogen source, its dietary inclusion rate must be carefully optimized to balance economic benefits with the preservation of adipocyte homeostasis and meat quality.

## Data Availability

The original contributions presented in the study are included in the article/supplementary material, further inquiries can be directed to the corresponding author.
